# Epigenetics and Oxidative Stress in Aging

**DOI:** 10.1155/2017/9175806

**Published:** 2017-07-20

**Authors:** Amy Guillaumet-Adkins, Yania Yañez, Manuel D. Peris-Diaz, Ines Calabria, Cora Palanca-Ballester, Juan Sandoval

**Affiliations:** ^1^CNAG-CRG, Centre for Genomic Regulation (CRG), Barcelona Institute of Science and Technology (BIST), Barcelona, Spain; ^2^Pediatric Oncology Unit, Hospital Universitari i Politècnic La Fe, Avda Fernando Abril Martorell, 46026 Valencia, Spain; ^3^Biomarkers and Precision Medicine Unit, Health Research Institute La Fe, Avda Fernando Abril Martorell, 46026 Valencia, Spain; ^4^Genomics Unit, Health Research Institute La Fe, Avda Fernando Abril Martorell, 46026 Valencia, Spain; ^5^Epigenomics Core Facility, Health Research Institute La Fe, Avda Fernando Abril Martorell, 46026 Valencia, Spain

## Abstract

Aging is a multifactorial process characterized by the progressive loss of physiological functions, leading to an increased vulnerability to age-associated diseases and finally to death. Several theories have been proposed to explain the nature of aging. One of the most known identifies the free radicals produced by the mitochondrial metabolism as the cause of cellular and DNA damage. However, there are also several evidences supporting that epigenetic modifications, such as DNA methylation, noncoding RNAs, and histone modifications, play a critical role in the molecular mechanism of aging. In this review, we explore the significance of these findings and argue how the interlinked effects of oxidative stress and epigenetics can explain the cause of age-related declines.

## 1. Introduction

Aging is a progressive loss of physiological integrity, leading to impaired function and increased vulnerability to death. It involves a very complicated physiological process, which as of today is still poorly understood. Scientific research has brought up many different theories trying to explain the aging problem, but none of them fully explain all of the aspects of this biological process. The most known and studied is the free radical theory of aging by Denham Harman, which identifies the accumulation of free radicals produced by the energetic metabolism of the mitochondria that end up causing cellular toxicity [1] and damage to the nuclear DNA [2], to cellular membrane structures [3], and to mitochondrial DNA (mtDNA) [4]. There are several evidences supporting that aging is also associated with epigenetic changes [5]. In the last few years, many efforts have been made to catalog the cellular and molecular hallmarks of aging and the interconnection between them. Herein, we give a comprehensive overview on how the combination of different epigenetic alterations and oxidative stress affects the process of aging.

## 2. The Epigenetic Machinery

Waddington first introduced the concept of epigenetics in 1939, “the actual interactions between genes and their products to phenotype into being” [6]. Then, Holliday, in 1987, redefined the term epigenetics as heritable changes in gene expression that are not due to alterations in the DNA sequence [7, 8]. Therefore, these heritable changes, regulated by different systems include DNA methylation (DNAm), noncoding RNAs, histone modifications, and variants [9]. These mechanisms have been shown to be indispensable in the regulation of tissue gene expression, X-chromatin inactivation, and genomic imprinting. All of these modifications put together create “the epigenetic landscape,” allowing the genome to display unique properties and distribution patterns in different cell types for its cellular identity [10]. DNA methylation was the first epigenetic modification discovered, and it is the best and most mechanistically understood. The enzymes that shape the DNA methylation patterns are the DNA methyltransferases (DNMTs), which are introduced onto the C5 position of cytosine residue, a methyl group (5mC) deriving from S-adenosylmethionine (SAM). In mammals, there are three types of enzymes, DNMT1, DNMT3a, and DNMT3b, which modify cytosine followed by a guanine residue, known as CpG dinucleotide. Even though DNA methylation is a stable epigenetic mark, it can be removed as a consequence of passive or active demethylation processes. Passive loss of methylation can be achieved through successive cycles of DNA replication in the absence of functional enzymes, such as DNMT1/UHRF1 [11], downregulation of the DNMT enzymes [12], DNMT cytosolic localization [13], and impairment of DNMT recruitment on DNA [14]. The active removal of 5mC has been shown to be through the formation of 5-hyroxymethylcytosine (5hmC), oxidized by the ten-eleven translocation (TET) enzymes. The oxidized products can then be processed directly by the TDG (thymine DNA glycosidase) generating a site that can be repaired by the BER machinery [15] or deaminated by AID deaminases (activation-induced cytidine deaminase), generating 5-hydroxymethyluracil (5hmU), which can also be excised by the TDG [16].

Eukaryotic DNA is packaged into chromatin, consisting of nucleosome units wrapping 147 bp of DNA around an octamer of four core histones (H2A, H2B, H3, and H4). The DNA bridging of two adjacent nucleosomes is the linker histone H1, termed linker DNA. Historically, chromatin has been classified as either euchromatin or heterochromatin, according to its compaction state, even though there is a spectrum of chromatin states, suggesting it to be a highly flexible macromolecule. Chromatin structure can be modified by writer, reader, and eraser chromatin enzyme complexes that can remodel the nucleosomes or modify the histones through posttranslational modifications (histone acetylation, phosphorylation, glycosylation, ubiquitylation, and SUMOylation), establishing different chromatin transcriptional states [17]. Lastly, noncoding RNAs (ncRNAs) can exert their regulatory function by acting as epigenetic regulators of gene expression and chromatin remodeling. Detailed mechanisms are still at a very early stage, but they are known to recruit different histone-modifying enzymes that recognize (read), add (write), remove (erase), and replace chromatin modifications. A bona fide example is the long noncoding RNA (lncRNA), XIST, the X-inactive-specific transcript, that coats one of the X chromosome by recruiting the polycomb repressive complex 2 (PRC2), triggering the heterochromatinization and transcriptional repression of the entire X chromosome [18, 19]. Therefore, understanding how the dynamics and the regulation of different epigenetic modifications are involved in the process of aging is of great interest.

## 3. Oxidative Stress

Oxidative stress is the disequilibrium between the reactive oxygen/nitrogen species (ROS and RNS) and the antioxidants, caused by a natural physiological process in the biological system, where the presence of these free radicals overpowers the scavenging mechanisms [20]. ROS are highly reactive molecules, which consist of diverse chemical species, including superoxide anion (O_2_^−^), hydroxyl radical (OH), and hydrogen peroxide (H_2_O_2_). The uncontrolled production of ROS will eventually interact with molecular structures, such as DNA, proteins, lipids, and carbohydrates, leading to an alteration of the metabolic pathway activity. This effect will cause molecular damage, that will eventually result in the pathogenesis of different diseases, such as cancer, neurodegenerative diseases, and diabetes, as well as aging [21]. The mitochondria are the main intracellular source of ROS generation, as a consequence of electron transfer during ATP production [22, 23]. Dysfunctional mitochondria leak electrons generating O_2_^.−^ as by-products, especially on the complex I (NADH dehydrogenase) and complex III (cytochrome bc 1 complex) [24]. Increased ROS production may also be caused by exogenous factors, such as radioactivity and ultraviolet irradiation. To prevent oxidative stress, cells are equipped with an antioxidative defense network, consisting of enzymatic and nonenzymatic mechanisms. These endogenous antioxidant enzymes are glutathione-S-transferase P1 (GTSP1), glutathione peroxidase, catalase, superoxide dismutase (SOD), sulfiredoxin, and peroxiredoxin [25]. As for the nonenzymatic mechanisms, these consist of a diversity of low molecular weight antioxidants which include glutathione and vitamins C, A, and E [26]. These both systems rely on each other to be effective, but when all these antioxidants are scarce, ROS increases and the normal redox state of the cell is altered, provoking oxidative stress, resulting in cellular damage.

## 4. The Link between Epigenetics and Oxidative Stress

Oxidative stress occurs as a consequence of ROS accumulation; this phenomenon increases with age, and it is accompanied by a decline in the cell repair machinery, which will eventually cause a wide range of DNA lesions leading to mutations as well as a disruption in the epigenetic state of the cell. Herein are some examples of how this tight interconnection interplays between the effect of oxidative stress and the epigenetic landscape. For example, ROS can influence the methylome through the formation of oxidized DNA lesions formed by hydroxylation of pyrimidines and 5-methylcytosine (5mC), which can interfere due to structural similarities with epigenetic signals related to 5-hmC [27]. ROS also affects DNA demethylation by DNA oxidation and TET-mediated hydroxymethylation [28]. ROS can indirectly modulate the activity of the epigenetic machinery since histone-modifying enzymes depend on intracellular levels of essential metabolites, such as Acetyl-CoA, Fe, ketoglutarate, NAD^+^, and S-adenosylmethionine, indicating that epigenetic changes are tightly linked to global cellular metabolism and energy levels of the cell [29]. Therefore, oxidative stress can globally influence the cell on multiple levels, from DNA and histones to histone modifiers, which will directly affect the epigenetic landscape of the cell.

## 5. Epigenetic Changes Associated with Aging

Epigenetic alterations represent one of the hallmarks of aging [5], by being an important mechanism behind the deteriorated cellular functions observed during aging. It is so that epigenetics serves as the missing link explaining why the pattern of aging is different between two identical twins [30]. The information encoded within our epigenome includes DNA methylation, chromatin remodeling, posttranslational modifications of the histone proteins, structural and functional variants of histones, and transcription of noncoding RNAs (ncRNAs). The combination of all of these different types of epigenetic information comprises the function and fate of all cells and tissues.

### 5.1. DNA Methylation

In aging, global reduction in DNA methylation and promoter hypermethylation of specific genes occurs [31, 32]. DNA hypomethylation takes place in transposable DNA repetitive elements, including Alu and LINE-1 elements, resulting in an increase retrotransposon activity and genomic instability [33]. Hypermethylation of specific CpG islands of regulatory genes of transcription [32], apoptosis [34], development, and differentiation [35] have also been described to be affected in aging. Bocklandt and collaborators identified the epigenetic pattern of three genes (*EDARDD*, *TOM1L1*, and *NPTX2*), that could accurately predict the physiological age, indicating a specific pattern of methylation in aging [36], among other studies that could also predict age through genome-wide methylation studies [37]. These sites underline the concept of epigenetic clock, which refers to specific sites that are consistently related to age across individuals. The enzymes involved in DNA methylation, such as DNMT1 and DNMT3a, are also altered during aging [38].

### 5.2. The Epigenetic Clock

The epigenetic drift refers to the modification of DNA methylation by age, and from this, the term epigenetic age uses DNA methylation levels to calculate the chronological age of cells and tissue samples [30]. As mentioned above, the epigenetic machinery maintains the DNA methylation during cell division by the DNMT enzymes, mainly DNMT1, DNMT3a, and DNMT3b in mammals. However, when the process fails, it leads to a loss of DNAm (hypomethylation) or gain of DNAm (hypermethylation) [39]. CpG methylation is probably one of the most widespread epigenetic modification monitored through the human genome to set up prediction/prevention strategies. One of the first studies in 2005 demonstrated how DNA methylation changes with age from monozygotic twins might impact in modifying the phenotype [30]. Further, other studies investigated and identified cytosines to predict the epigenetic age of specific tissues [36, 40, 41]. In blood, an epigenetic signature to estimate aging, related to three CpG sites, was established. These are located in the genes integrin, alpha 2b (*ITGA2B*), aspartoacylase (*ASPA*), and phosphodiesterase 4c (*PDE4C*). The findings surrounding allowed performing a regression model that showed a mean absolute deviation from the chronological age of less than five years [40]. In saliva, Bocklandt et al. [36] recognized 88 CpG sites near 80 genes, which revealed higher levels of 5mC correlated with age (*q* value < 0.05). Gene ontology analysis deciphered enrichment for genes involved in age-related diseases, cardiovascular, genetic, and neurological diseases, and genes involved in molecular transport. To predict the age of a person based on a biological sample, a multivariate linear regression was built based on the methylation of only two cytosines (Edar-associated death domain (*Edaradd*) and neuronal pentraxin II (*NPTX2*) genes), that covered 73% of the variance in age, with an average accuracy of 5.2 years. In blood, Hannum et al. [41] introduced a specific DNAm-based age predictor formed by a large compilation of methylation data (656 blood samples aged 19–101 years). The model included 71 CpG sites with age-related genes displaying an error prediction of 4.9 years. Many of those epigenetic markers were implicated in aging, metabolic activity, and longevity. The gene somatostatin (*SST*), which regulates the endocrine and nervous system function, and its involvement in Alzheimer's disease, and the transcription factor *KLF14* involved in metabolism were linked to model markers.

However, these specific tissue models may not achieve a proficient predictive result since their accuracy was validated by limited specific datasets, although leading to a high accuracy in age-associated changes in specific cell and tissue type (e.g., blood or saliva). To overcome this issue, Horvath established an independent tissue and cell-type predictor of age [42]. The model, which was built by means of almost 8000 noncancer samples including 51 different tissues and cell types and based on 353 CpGs, led an average accuracy of 5 years. Ingenuity pathway analysis showed that the 353 clock CpGs were enriched for genes involved in cell death/survival, cellular growth/proliferation, organismal/tissue development, and cancer. Horvath's epigenetic clock avoids confounding by age-associated changes in tissue-specific, but it was useless for diseases promoted by mitotic activities like cancer [39]. Knight et al. [43] developed an epigenetic clock model to estimate gestational age at birth, examining DNAm in cord blood using 148 CpGs and 1434 DNAm data. The predictor developed showed a comparable accuracy to the established clinical methods (median error of 1.24 weeks) but affordable in cases where clinical measurements are not available. Compared to Horvath's predictor [42], higher predictive ability was found for the model proposed. Of interest, what implicates differences between epigenetic age or DNAm-age and chronological age is discussed in many studies. In this context, the term “age acceleration” refers to the deviation observed between them, which have been proved to assess the biological age and correlated with the state of some diseases, like HIV-1 and Down syndrome [39]. In Knight et al. [43], through the residual of the fitted linear model, the authors defined a similar parameter to DNAm-age, termed as gestational age acceleration. It was used as a biomarker of perinatal health, which is associated with birthweight and risk of mortality. The epigenetic age acceleration has also been proposed as indicative for life expectancy [44]. Lin et al. [45], through survival analysis and using 99 CpGs, identified 11% greater mortality risk for five-year higher age prediction when applied the 99-CpG model for the Lothian Birth Cohort 1921 (LBC1921). Further, several CpGs were associated with genes related with life expectancy, such as with *PDE4C*. However, in the Lothian Birth Cohort 1936 (LBC1936), where there are lower number of deaths, 99-CpG model could not assess CpG correlation with mortality, while age predictions models by Hannum et al. [41] and Horvath [42] were able to. In this line, Chen et al. [46] in a meta-analysis demonstrated the evidence that the epigenetic age acceleration, that only requires the measurement of DNA methylation, is related with a cell-intrinsic epigenetic aging process. They informed how age acceleration implicates higher mortality risk and is independent of changes in blood cell composition during aging. Moreover, epigenetic age captures processes of biological age, additionally to risk factors that have large influence on mortality. Thus, to assess the biological age, effects promoted by either endogenous or exogenous factors should be considered, besides the chronological age. Future studies will be necessary to gain molecular mechanistic knowledge about epigenetic processes and how these changes affect over aging phenotype. To conclude, epigenetic biomarkers will increase our understanding of aging in health and disease and thus will improve the clinical evaluation and treatment of patients.

### 5.3. Histone Modifications

Histones are subjected to a wide variety of posttranslational modifications (PTM) that have a severe impact on the global structure of the chromatin, influencing gene expression, genome stability, and replication. Therefore, this array of modifications orchestrates the functional responses that will affect all biological processes, including aging. In senescence, an imbalance of activating and repressive histone modifications occurs. Histone methylations, H3K4me3, and H3K27me3, which are epigenetic modifications linked to transcription, have been related to lifespan regulation. For example, the inhibition of the methyltransferases, ASH-2 and SET-2, has been associated with a global reduction of levels of H3K4me3, increasing life expectancy. On the other hand, inhibition of demethylase RBR-2 reduces longevity [47, 48]. The global pattern of histone methylation differs among different organisms, due to differential aging process, but the trend is that there is an increase in the appearance of activating histone methylation marks during aging affecting the compaction of chromatin in aging cells [49]. Other examples are studies done in *C. elegans* on histone deacetylase SIR-2, responsible for the acetylation of H4K16, which has been associated to increase longevity [50, 51]. On the contrary, the homolog SIRT-1 is decreased in aging probably due to an increase of ROS [52], causing a reduced heterochromatic silencing leading to an altered gene expression in aging [53]. Importantly, this provides a compelling evidence on how the epigenetic role plays in the lifespan regulation.

### 5.4. Noncoding RNAs

Noncoding RNAs (ncRNAs), both short ncRNAs (mostly microRNAs) and long ncRNAs affect aging by controlling gene expression transcription and posttranscription in a myriad of ways. The best-characterized example of roles of miRNAs during aging comes from studies in *C. elegans*. The most remarkable one is microRNA *lin-4* [54] that regulates aging and proaging target microRNA *lin-14* [55]. Loss of expression of *lin-4* shortens life span, and overexpression of *lin-4* does not, while knocking down *lin-14* extends life span. During aging, differential expression of miRNA is also evident [56] and lncRNAs are also deregulated in aging-related diseases [57]. A bona fide example is *H19* lncRNAs, which controls the imprinting of a conserved cluster of *H19* and *IGF2.* Loss of imprinting of the *IGF2-H19* leads to the increase of the *IGF2*, associated with age-related diseases [58].

## 6. Oxidative Stress and Aging

**Figure 1:**
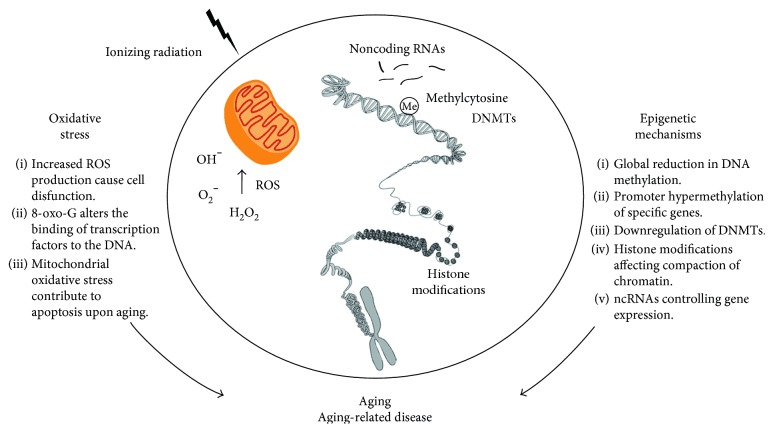
Epigenetic mechanisms and oxidative stress (OS) related to aging. Oxidative stress caused by either endogenous or exogenous factors gives rise to increased levels of reactive oxygen species (ROS), mitochondria acting as a main source of production. Uncontrolled production of ROS is involved in aging and aging-related diseases. Besides, ROS is an activity-modulated factor of epigenetic machinery. Epigenetic changes, for instance, global reduction in DNA methylation or hypermethylation of specific genes, among others, are also linked with aging.

The consequences of oxidative stress have raised many theories trying to explain the aging phenomena; one of these is the free radical theory of aging which postulates that aging results from the accumulation of deleterious effects caused by free radicals [1]. In agreement with this theory, increased ROS production by mitochondria is frequently detected in aged tissues [59], and it has been suggested as the main cause of aging [60]. Several other studies have also reported that increased oxidative damage in cells is associated with aging [61, 62]. This effect leads to the accumulation of damage in lipids, nucleic acids, proteins, and carbohydrates, causing cellular dysfunction and making the body more prone to harmful external agents. Since mitochondria are the major producer of ROS in mammalian cells, one of the common oxidative lesions, 8-oxo-7,8-dihydro-2′-deoxyguanosine (8-oxo-dG), detected a higher level in mtDNA than in nuclear DNA, suggesting that mtDNA is more susceptible to oxidative damage [63–65]. The mitochondrial theory of aging, extended from the free radical theory, postulates that oxidative damage generated during oxidative phosphorylation of mitochondrial macromolecules such as mtDNA, proteins, or lipids is responsible for senescence [66]. Furthermore, the mitochondria play a critical role in the regulation of apoptosis; therefore, age-related mitochondrial oxidative stress contributes to apoptosis upon aging [67]. Growing experimental evidence shows beneficial effects of mitochondrial-targeted antioxidants in aging [68]. They have been shown to confer greater protection against oxidative damage in the mitochondria than untargeted cellular antioxidants, due to the ability to cross the mitochondrial phospholipid bilayer, and eliminate ROS at the heart of the source [69–71] (Figure 1).

## 7. Aging-Related Diseases as a Consequence of Epigenetics and Oxidative Stress

Aging causes impairment of the epigenetic landscape, as well as the increase of oxidative stress in the cell that will eventually contribute to the development of the disease. Progeroid syndromes are a group of systemic diseases that greatly resemble physiological aging, being a powerful tool to study the physiological process of aging. The most known premature aging diseases are the Hutchinson-Gilford Progeria syndrome (HGPS; OMIM: 176670) and Werner syndrome (WS; OMIM: 277700). HGPS is caused by a mutation in the *LMNA* gene, which encodes two protein products (lamin A and lamin C), representing major constituents of the inner nuclear membrane lamina [72]. This mutation leads to an abnormal version of the lamin A protein called progerin. On the other hand, WS is caused by mutations in the *WRN* helicase gene [73], involved in the DNA repair pathway. HGPS is particularly susceptible to DNA damage induced by ROS, showing an impaired capacity to repair DNA damage [74]. In these syndromes, epigenetic and chromatin structures are also affected. Aberrant DNA methylation profiles occur by gaining methylation in hypomethylated regions and losing methylation in hypermethylated regions [75]. A loss of heterochromatin is also observed due to the absence of *WRN* in WS [76], and the accumulation of progerin in HGPS [77]. In HGPS, there is a loss of epigenetic mark H3K27me3 on the inactive X chromosome as a consequence of a downregulation of the EZH2 methyltransferase enzyme [78]. Another example of disease where both epigenetics and oxidative stress are linked to the pathogenesis occurs in the respiratory system, which is continually exposed by endogenous (mitochondrial respiration) and exogenous (air pollutants, noxious gasses, and cigarette smoking) sources of oxidants. This accumulation of ROS directly decreases the functionality of lung cells. This is very evident in the case of smokers and obstructive pulmonary disease (COPD), where an increase of ROS leads to a deregulated expression of proinflammatory genes and the reduction of the enzymatic activity of HDAC2 [78, 79]. This histone deacetylase delays cellular senescence by negatively regulating prosenescent genes, such as p21 and p16 [78]. Therefore, the reduction of the histone deacetylase may accelerate cellular senescence in COPD. The nervous system is also susceptible to oxidative stress. In Alzheimer's disease, one of the earliest events in the pathogenesis of the disease is the oxidative DNA damage [80]. The most common oxidative lesion is the oxidation of guanine to 8-oxo-dG, which alters the binding of transcription factors to the DNA [81]. In cardiovascular diseases, there is a growing evidence of the role that epigenetic modifications and oxidative stress play in the pathogenesis of these diseases. The production of nitric oxide (NO) by nitric oxide synthases (NOSs) has an important cardioprotective role against cardiovascular disease by regulating blood pressure and vascular tone and inhibiting platelet aggregation and leukocyte adhesion, but when NO interacts with superoxide, this forms peroxynitrite decreasing its activity, enhancing oxidative stress. Inactivation of NO by ROS is responsible for the endothelial dysfunction, contributing to cardiovascular diseases. In cancer, oxidative stress is also implicated. Oncogenic-driven cancer cells generate increased ROS as by-products of their metabolism to maintain tumorigenicity [82]. High levels of ROS induce death; however, cancer cells over bypass this by upregulating intracellular antioxidant proteins to maintain ROS levels to allow protumorigenic signaling without resulting in cell death [83–86]. In non-small-cell lung cancer (NSCLCs), SOD1 (superoxide dismutase 1) is expressed at high levels. The function of the enzyme is to maintain low levels of superoxide in the cytosol, protecting the cell from oxidative stress and subsequent cell death. By targeting this enzyme with a small molecule, ATN-224 reduced tumor growth, suggesting a potential clinical application in these types of adenocarcinomas [87].

## 8. Conclusions

Herein, we review how the interlinked effects of oxidative stress and epigenetic changes affect the process of aging. These findings open new horizons in the comprehension of the molecular basis of aging, such as the production of ROS and its effects on the epigenetic machinery. Therefore, it will only be the complete understanding of this molecular process of aging and aging-associated diseases that will help us tackle this natural physiological process to a healthier aging.
